# Conditional random pattern model for copy number aberration detection

**DOI:** 10.1186/1471-2105-11-200

**Published:** 2010-04-22

**Authors:** Fuhai Li, Xiaobo Zhou, Wanting Huang, Chung-Che Chang, Stephen TC Wong

**Affiliations:** 1 Center for Bioengineering and Informatics, Department of Radiology, The Methodist Hospital Research Institute, Weill Cornell Medical College, Houston, TX 77030, USA; 2 Department of Pathology, The Methodist Hospital Research Institute, Weill Cornell Medical College, Houston, TX 77030, USA

## Abstract

**Background:**

DNA copy number aberration (CNA) is very important in the pathogenesis of tumors and other diseases. For example, CNAs may result in suppression of anti-oncogenes and activation of oncogenes, which would cause certain types of cancers. High density single nucleotide polymorphism (SNP) array data is widely used for the CNA detection. However, it is nontrivial to detect the CNA automatically because the signals obtained from high density SNP arrays often have low signal-to-noise ratio (SNR), which might be caused by whole genome amplification, mixtures of normal and tumor cells, experimental noise or other technical limitations. With the reduction in SNR, many false CNA regions are often detected and the true CNA regions are missed. Thus, more sophisticated statistical models are needed to make the CNAs detection, using the low SNR signals, more robust and reliable.

**Results:**

This paper presents a conditional random pattern (CRP) model for CNA detection where much contextual cues are explored to suppress the noise and improve CNA detection accuracy. Both simulated and the real data are used to evaluate the proposed model, and the validation results show that the CRP model is more robust and reliable in the presence of noise for CNA detection using high density SNP array data, compared to a number of widely used software packages.

**Conclusions:**

The proposed conditional random pattern (CRP) model could effectively detect the CNA regions in the presence of noise.

## Background

Detection of copy number aberrations (CNA) using single nucleotide polymorphism (SNP) array data or Array comparative genomic hybridization (CGH) data is becoming important in disease pathogenesis analysis [1-6]. For example, CNA may result in suppression of anti-oncogenes and activation of oncogenes, which would cause certain types of cancers [1,7,8]. Disease related CNAs not only indicate the molecular-level pathogenesis, but also can be used as biomarkers for diagnosis. For example, Myelodysplastic syndromes (MDS) are a group of clonal hematopoietic disorders, which are considered as clonal stem cell diseases characterized by peripheral cytopenias (anemia, neutropenia, and/or thrombocytopenia) with normocellular or hypercellular marrow and bilineage or trilineage dysplasia [9-13]. Early diagnosis with appropriate treatment may lead to improved prognosis, however, there is no accurate diagnosic method at the early stage of MDSs because the morphological appearances are highly variable and not specific to the MDSs [9-13]. Using the high density SNP arrays, the molecular-level biomarkers of MDSs may be detected, and are helpful for the MDS early diagnosis and treatment.

To detect the CNA regions using high density SNP arryas, automated SNP array analysis method is needed. However, it is nontrivial to detect the CNA automatically because the signals obtained from high density SNP arrays often have low SNR values, which may be caused by whole genome amplification, mixture of normal and tumor cells, experimental noise and other technical limitations. With the reduction in SNR, many false CNA regions are often detected and true CNA regions are missed. Thus, more sophisticated statistical models are needed urgently to make the CNAs detection robust and reliable using the signals with low SNR, although a number of software packages have been developed for the SNP array analysis. For Affymetrix SNP array, the widely used software packages are Genotyping Console [14], GEMCA [15], CNAG [16,17] and dChip [18,19] and Birdsuite [20,21]. For Illumina SNP array, PennCNV [22,23], QuantiSNP [24,25], GenoCN [26,27], SOMATICs [28,29] and OverUnder [30,31] have been developed. For array-CGH data, aCGH (Microarray-based comparative genomic hybridization) [32], CLAC (Clust Along Chromosomes) [33], CBS (Circular binary segmentation) [34,35] and GLAD (Gain and loss analysis of DNA) [36], and many other CNA detection algorithms have been developed [37]. Usually there are two types of copy number analysis: one is the CNA, the other is copy number variation (CNV) analysis. The CNVs naturally happens in normal tissue and are inheritable, while the CNA are acquired somatic alterations and often observed in disease tissues, which also tend to be longer and more densely occur in the genome [23,27]. Most of the abovementioned software packages could detect both CNVs and CNAs, whereas some of them may incorporate more information to improve their performance specifically for CNV or CNA detection. For example, the SOMATICs and GenoCNA incorporate the normal tissue contamination information for better CNAs detection.

GEMCA is a good software package for copy number variants detection in HapMap data, which can combine both Nsp and Sty arrays to detect small copy number variant regions. It is stated in the website of this software that it is not suitable for cancer analysis that has much larger copy number changes, and the new software programs are being developed for cancer copy number analysis [3,38,39]. For Genotyping Console, CNAG [17] and dChip [19,40], although different pre-processing techniques (e.g. normalization, scaling and feature extraction) are used, they all use HMM framework [41] in the second tier to infer the copy number. However, the major limitation of the HMM framework lies in the simple assumption that the current state is only determined by the immediate previous state and the current observation [41]. Due to this assumption, the noisy SNP array data often results in inaccurate copy number inference. In Birdsuite software package, the Birdseye method, which is a HMM, is implemented to find CNV regions [20,21]. The QuantiSNP, PennCNV and GenoCN all make use of the HMMs on the two dimensional data: Log R Ratio (LRR) and B allele frequency (BAF) [23,24]. Compared to QuantiSNP, PennCNV improved the transition probabilities in HMM, BAF distribution, accuracy of likelihood of copy number genotype modelling, and added the family-based analysis [23]. In the GenoCN software, the CNV and CNA detections are processed by two different modules: GenoCNV and GenoCNA [27]. The GenoCNA incorporates the normal tissue contamination and genotype data from normal tissue to improve the CNA detection accuracy [27]. SOMATICs uses the two-band pattern of BAF to detect the CNAs of samples that mix the normal and tumor cells [28]. OverUnder detects the CNA by dividing the 2D square of the B allele frequency (BAF) and the Log R Ratio (LRR) into different regions that correspond to the loss, gain, amplification [31]. aCGH uses the unsupervised HMM. CBS (circular binary segmentation) makes use of the maximum of a likelihood ratio statistic recursively to separate SNP sequence into small segments. In GLAD method, an adaptive weights smoothing procedure is used to estimate the means of sequence segments. In CLAC the hierarchical clustering method is used to detect the CNA regions. CBS, GLAD and CLAC only estimate the means of sequence segments, rather than give the exact copy number of each segment.

In this study, we present a novel conditional random pattern (CRP) model for CNA detection, in which more contextual information of neighboring SNP loci is considered, compared to HMM, to suppress the noise and improve the accuracy of CNA detection. Specifically, in the CRP model, the copy number of a SNP locus is not only determined by the copy numbers of its two immediate neighboring SNP loci, but also by a continuous segment of observations (log2-ratio features), thus allows us to employ more contextual cues. The rest of this paper is organized as follows. The details of the CRP model are described in Section 2. Section 3 provides the experimental validations, and the discussion and conclusions are presented in Section 4.

## Results

To validate the proposed CRP model, we compared the proposed CRP model with dChip, CNAG and PennCNV-Affy, and four widely used copy number inference software packages: aCGH, CBS, CLAC, and GLAD, using both simulated data and real data.

### Validation data

To validate the proposed CRP model, three real data sets were employed. The first one is from the MDSs samples in our laboratory. 12 cryopreserved bone marrow samples from MDS patients were analyzed. Please see the detailed samples' information in the additional file 1. The second one is the array-CGH data used in Lai's 2005 paper [37]. We also downloaded the HapMap samples' 500 K SNP array data from the Affymetrix website http://www.affymetrix.com/support/technical/sample_data/500k_hapmap_genotype_data.affx. Two HapMap samples, NA10851 (as reference sample) and NA18515 (as test sample) were used to test the CRP model, in which some CNA regions were validated by quantitative PCR in [3,4].

Since there is no ground truth of the CNA regions in these data, it is difficult to quantitatively measure the performance of different software packages. Therefore, two more simulated data sets were created. We simulated Affymetrix GeneChip Human Mapping 500 K Array data in the Affymetrix's CEL file format, which is based on the real 500 K SNP arrays of HapMap samples from the Affymetrix website, therefore both dChip and CNAG (and other software packages) can process them. The simulation process of these data is as follows. First, we randomly selected three arrays as the normal reference samples: "NA10851_FinNsp_vR1_579813_A1_1_SC2", "NA12812_FinNsp_vR1_579813_B2_1_SC6" and "NA18605_FinNsp_vR1_579548_D5_1_SC3". Secondly, we randomly set one CNA region in each chromosome, and a total of 22 CNA regions were obtained (Chromosome X was not set). The length of these CNA regions varies from 4 to 100 SNPs uniformly. Thirdly, for each reference sample and certain noise level, two simulated arrays are generated, one for copy number deletion (one copy), and the other for copy amplification (three copies). The mismatch probes are used as the background to estimate the simulated intensities of the corresponding perfect match probes in these CNA regions. The intensity of probes outside the CNA regions remains unchanged. Then the Gaussian noises are generated and added to all probes, which follow a Gaussian distribution, *N*(0, *σ*), where the standard deviation of noise *σ *is proportional to the probe intensity *y*. The signal to noise ratio (SNR) *SNR = y/σ *varied from 5, 2 to 1.25, to simulate different noise levels. A total of 18 samples were simulated based on the three selected HapMap samples (2 simulations per SNR level per sample * 3 SNR levels * 3 HapMap samples).

We also simulated log2-ratio sequences. For each noise level, we simulated 100 log2-ratio sequences, and each sequence contains 300 log2-ratio data points. Four CNA regions with 5, 10, 20 and 40 length were created. In the CNA regions, the mean was set as 0.4, and outside the CNA regions the mean was zero. Then the Gaussian noise *N*(0, *σ*) was added. Three noise levels were considered, and the SNR (0.4/*σ*) varied from 2, 1.3 to 1.

### Validation metrics

To measure the performance of copy number inference software packages, seven metrics were used: snp-precision (sp), snp-recall (sr), region-precision (rp), region-recall (rr), hybrid-precision (hp), hybrid-recall (hr), and f-score (f). Given a contingency Table, as seen in Table 1, the seven metrics are defined as follows.

 and .

The snp-precision and snp-recall measure the accuracy in the single SNP level, which indicate how many SNPs in all the CNA regions are detected or missed. The CNA region based metrics indicate how many single CNA regions are detected (at least one SNP in the CNA region is detected) or missed (all the SNPs in the CNA region are missed) without considering the length of the CNA regions. The hybrid metrics combine the single SNP and CNA region based metrics together to generate the f-score to examine the overall performance of a software package.

**Table 1:** The Contingency Table

Ground Truth Prediction	CNA SNP Loci	Normal SNP Loci	CNA Regions	Normal Regions
CNA SNP Loci				

Normal SNP Loci				

CNA Regions				

Normal Regions				

### Comparison with dChip, CNAG and PennCNV-Affy

To evaluate the performance of the CRP model, we compared it with a number of widely used software packages: dChip, CNAG and PennCNV-Affy. In August 2009, PennCNV provided the PennCNV-Affy protocol to calculate the LRR and BAF signals from Affymetrix SNP arrays, and then make the CNA detection.

#### Results on simulated 500 K SNP array data set

**Figure 1:**
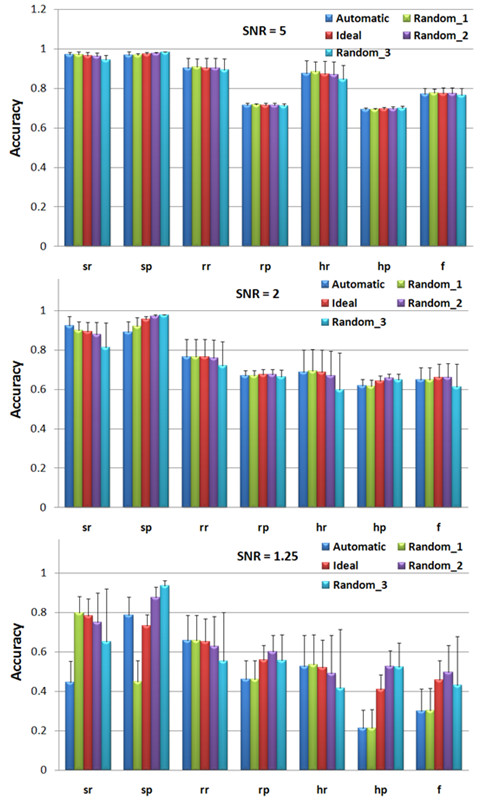
**CNA detection performance of CNAG with five different parameter settings on the simulated Affymetrix 500 K SNP array under different SNR values**.

**Figure 2:**
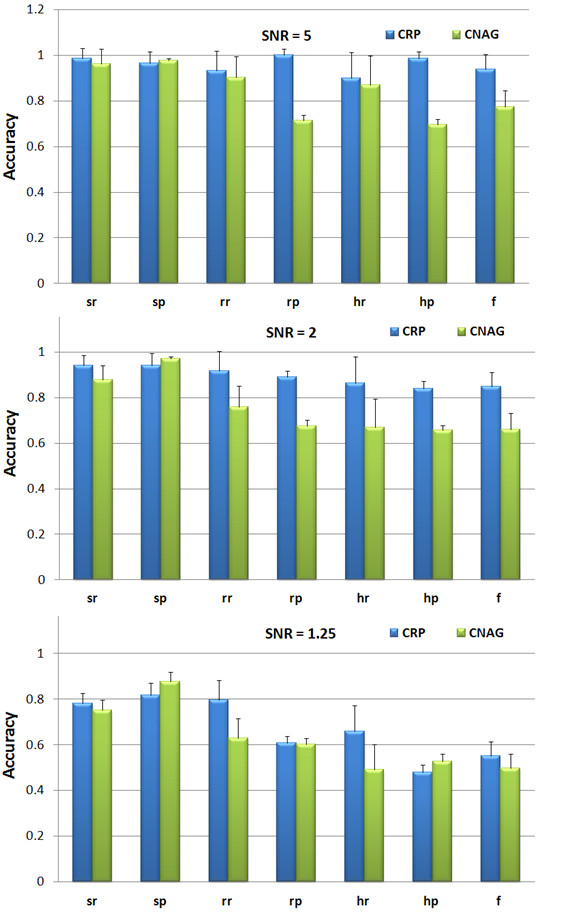
**CNA detection performance comparison between the CRP model and in the CNAG with the optimal parameter setting on the simulated Affymetrix 500 K SNP array samples under different SNR values**.

The proper comparisons between different algorithms are not trivial because of different possible parameter settings in each algorithm. In CNAG, users can manually set the means of the HMM for each copy number status. To make the comparison fair, we tried to find the best performance of the CNAG by testing a few different parameter settings. Then we compared the CRP model with the best performance of CNAG. Since we simulated the three-copy amplifications and one-copy deletions in the simulated 500 K SNP array data, we only need to set the means of three-copy, M_CN3 _= 0.38, and one-copy, M_CN1 _(the mean of two-copy is set as zero) in CNAG. We tested following five different parameter settings of HMM in CNAG: 1) 'Automatic' - the parameters are set by the CNAG automatically. 2) 'Ideal' - (M_CN3 _= 0.38, M_CN1 _= -0.45). We estimated the means in the 'Ideal' by calculating the means of the three-copy amplifications and one-copy deletions in the simulated data respectively. 3) 'Random_1' - (M_CN3 _= 0.38, M_CN1 _= -0.42). 4) 'Random_2' - (M_CN3 _= 0.4, M_CN1 _= -0.5). 5) 'Random_3' - (M_CN3 _= 0.45, M_CN1 _= -0.55). Figure 1 shows the performances of CNAG on the simulated 500 K SNP arrays with the five different parameter settings. As we can see, the CNAG generated 'best' results in the 'Random_2' settings. Then we compared the CRP performance with the 'best' results of the CNAG. The detailed CNA detection results of the CRP model and that of the CNAG, with the 'Random_2' parameter setting, are provided in Table 2 and Table 3 respectively. Figure 2 provides the performance comparison between the CRP model and CNAG with optimal parameter setting. Obviously, the CRP model outperforms the CNAG in all three SNR levels and all 18 simulated 500 K SNP samples significantly.

**Figure 3:**
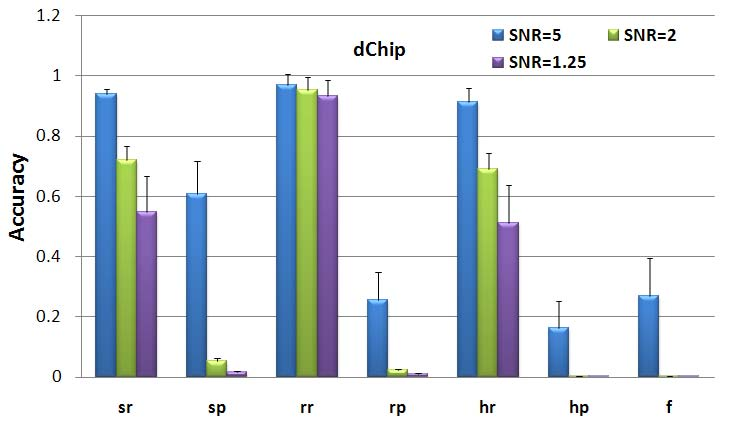
**CNA detection performance of dChip on the simulated Affymetrix 500 K SNP array under different SNR values**.

**Figure 4:**
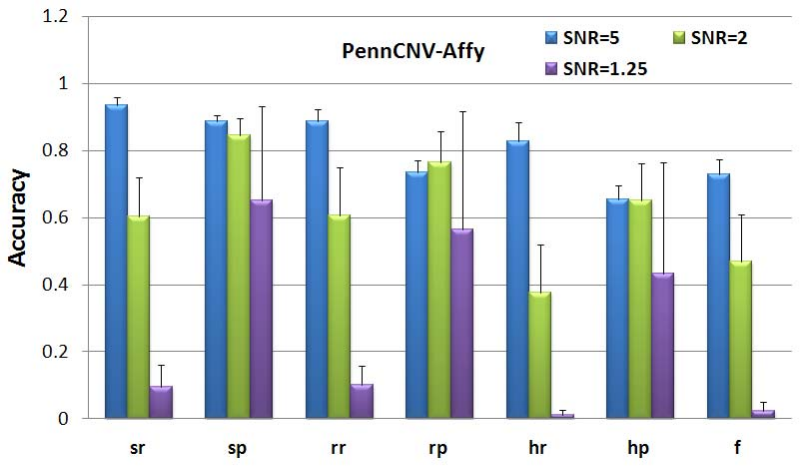
**CNA detection performance of PennCNV-Affy on the simulated Affymetrix 500 K SNP array under different SNR values**.

Since the parameters of HMM in dChip are determined automatically and are not user accessible [19,42], we evaluated the dChip using its default parameter setting. For PennCNV, we followed the user guide to evaluate its performance [22,23]. The comparison results with dChip and PennCNV-Affy are provided in Figures 3 and 4 respectively. From the high 'recall' and low 'precision' rates of dChip results, we concluded that dChip detected many false CNAs, as seen in Figure 3. The PennCNV-Affy works well on samples with high SNR value, while its accuracy decreases dramatically with the reduction in SNR, as seen in Figure 4. The low 'recall' and the high 'precision' rates indicate that the PennCNV-Affy is not sensitive to small CNAs. Through the comparisons, we conclude that the CRP model is more robust to noise and improves the accuracy of CNA detection significantly compared with the HMMs implemented in CNAG, dChip and PennCNV-Affy.

**Table 2:** CNA detection results of CRP model on the simulated SNP arrays

SNR	Samples	sr	sp	rr	rp	hr	hp	f
5.00	NA10851	0.986	0.972	0.955	1.000	0.928	0.986	0.956
		
		0.983	0.969	0.955	1.000	0.925	0.983	0.953
	
	NA12812	0.987	0.961	0.909	1.000	0.874	0.987	0.927
		
		0.984	0.962	0.909	1.000	0.875	0.984	0.926
	
	NA18605	0.987	0.961	0.909	1.000	0.874	0.987	0.927
		
		0.982	0.959	0.955	1.000	0.915	0.982	0.947

2.00	NA10851	0.945	0.964	0.955	0.913	0.920	0.863	0.890
		
		0.925	0.944	0.909	0.833	0.858	0.771	0.812
	
	NA12812	0.971	0.919	0.909	0.952	0.835	0.925	0.878
		
		0.975	0.919	0.909	1.000	0.835	0.975	0.900
	
	NA18605	0.909	0.969	0.909	0.800	0.881	0.727	0.797
		
		0.919	0.942	0.909	0.840	0.857	0.772	0.812

1.25	NA10851	0.801	0.914	0.864	0.583	0.790	0.467	0.587
		
		0.771	0.858	0.864	0.639	0.741	0.493	0.592
	
	NA12812	0.814	0.808	0.773	0.633	0.625	0.516	0.565
		
		0.731	0.775	0.864	0.579	0.670	0.423	0.519
	
	NA18605	0.854	0.912	0.818	0.704	0.746	0.601	0.666
		
		0.717	0.644	0.591	0.517	0.380	0.371	0.376

Key: Seven metrics were used: snp-recall (sr), snp-precision (sp), region-recall (rr), region-precision (rp), hybrid-recall (hr), hybrid-precision (hp) and f-score (f).

**Table 3:** CNA detection results of HMM (in CNAG) on simulated SNP arrays

SNR	Samples	sr	sp	rr	rp	hr	hp	f
5.00	NA10851	0.9718	0.9857	0.9091	0.7143	0.8834	0.7041	0.7836
		
		0.98	0.9709	0.9545	0.7241	0.9355	0.703	0.8028
	
	NA12812	0.9376	0.9803	0.8182	0.6923	0.7672	0.6787	0.7202
		
		0.9659	0.967	0.9091	0.7143	0.8781	0.6907	0.7732
	
	NA18605	0.9459	0.9841	0.8636	0.7143	0.8169	0.7029	0.7556
		
		0.98	0.9731	0.9545	0.7241	0.9355	0.7047	0.8038

2.00	NA10851	0.8918	0.9768	0.7727	0.68	0.6891	0.6642	0.6764
		
		0.92	0.9583	0.7727	0.68	0.7109	0.6517	0.68
	
	NA12812	0.7765	0.9836	0.6364	0.6364	0.4941	0.6259	0.5523
		
		0.9435	0.9686	0.9091	0.7143	0.8578	0.6919	0.7659
	
	NA18605	0.8235	0.979	0.6818	0.6667	0.5615	0.6527	0.6037
		
		0.9071	0.9674	0.7727	0.68	0.7009	0.6578	0.6787

1.25	NA10851	0.5247	0.8352	0.3636	0.4444	0.1908	0.3712	0.252
		
		0.9129	0.8509	0.7727	0.6538	0.7055	0.5563	0.6221
	
	NA12812	0.6165	0.9758	0.5455	0.6	0.3363	0.5855	0.4272
		
		0.8518	0.836	0.7273	0.64	0.6195	0.5351	0.5742
	
	NA18605	0.7718	0.9061	0.6364	0.6087	0.4911	0.5515	0.5196
		
		0.8235	0.8516	0.7273	0.6667	0.5989	0.5677	0.5829

Key: Seven metrics were used: snp-recall (sr), snp-precision (sp), region-recall (rr), region-precision (rp), hybrid-recall (hr), hybrid-precision (hp) and f-score (f).

#### Results on the real MDS data sets

**Figure 5:**
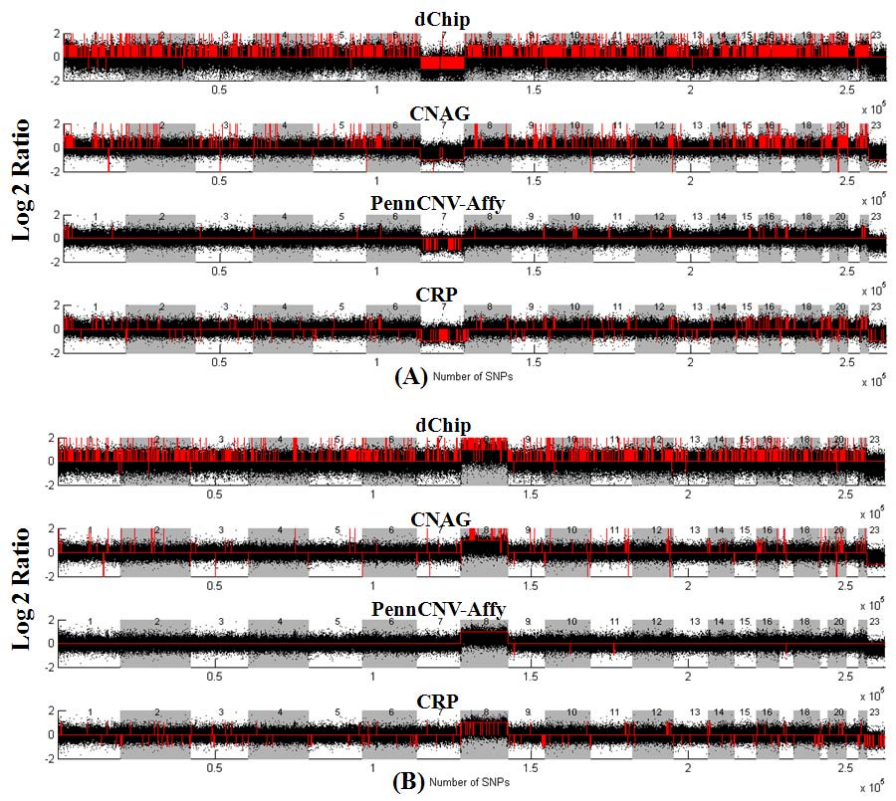
**CNA detection comparison among dChip, CNAG, PennCNV-Affy and CRP model on the real MDSs samples**. The black color dots denote the log2-ratio values, and the red color lines indicate the inferred copy number.

We applied the dChip, CNAG, PennCNV-Affy and CRP model to the MDSs Affymetrix 500 k SNP arrays. For both dChip and PennCNV, the default settings were used. For CNAG, we tested a few different parameter settings, and the one with best performance on the pre-known monosomy and trisomy regions is selected. Twenty SNP arrays of normal tissue DNA samples from the buccal and lymphoid tissues of ten MDS patients were used as the reference set. Figures 5 provide the CNA detection results of these four software packages on two MDS samples. The top, second, third and last lanes in Figure 5 present the results of dChip, CNAG, PennCNV and CRP model respectively. In sample 1, the Chromosome-7 has only single copy, and in sample 2, the Chromosome-8 has three copies as determined by conventional cytogenetic study. Obviously, dChip detects many false positive CNAs in both samples, and infers most of SNPs wrongly as four copies. Both dChip and PennCNV-Affy miss many deletion regions in Chromosome 7 of sample 1, and PennCNV-Affy works well on the Chromosome-8 of sample 2, however, it looks not sensitive to the small CNA regions (only few deletions are detected). After parameter adjusting manually, CNAG works well on the monosomy and trisomy Chromosomes. However, CNAG detects very few copy number deletions and some CNA regions with only one SNP. Low SNR of log2-ratio signals is one reason of these errors. Compared with dChip, CNAG and PennCNV-Affy, CRP model makes use of more contextual cues, and generates more accurate results. We identified some MDSs related regions, 7q34 (deletion) and 7p14.2 (gain), which were missed by dChip, CNAG, PennCNV-Affy. Additionally, a copy number deletion region carrying FOXP2 gene located at 7q31.1 was detected using CRP model in another case, while it was also missed by dChip, CNAG and PennCNV-Affy. This region was confirmed by quantitative PCR, as seen in the additional file 1[43].

### Comparison with aCGH, CBS, Clac and Glad

A number of CNA detection algorithms have been developed for array CGH data [37]. To further evaluate the performance of the CRP model, we compared the CRP model with four widely used CNA detection methods: aCGH [32], CLAC [33], CBS [34,35] and GLAD [36].

#### Results on simulated log2-ratio sequences

**Figure 6:**
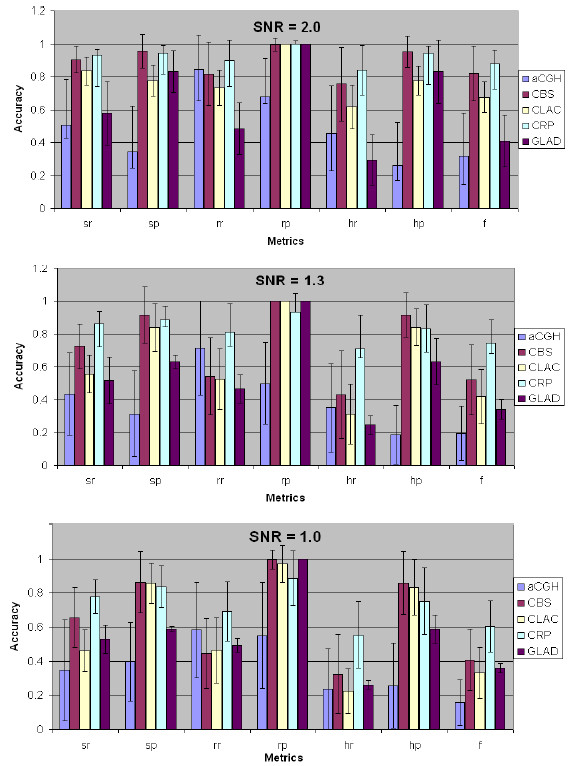
**CNA detection performance comparison among the CRP model, aCGH, CBS, CLAC and GLAD software packages**.

The average performances of the CRP model, aCGH, CBS, CLAC and GLAD software packages are provided in Figure 6. In terms of single-, region- and hybrid-precision, CRP, CBS and CLAC have similar performance, in which fewer false CNA regions were wrongly detected. However, they missed some small CNA regions, as seen by the recall-metric results in Figure 6. The results of aCGH were not good. aCGH software package used the unsupervised HMM, in which all the parameters of the HMM will be estimated based on the observation data [32]. However, the parameter estimation method is sensitive to noise, and then often causes inaccurate CNA detection results. In terms of single-, region- and hybrid-recall, CRP model generates better results than the other software packages, which means more CNA regions were missed by the other software packages, especially the small CNA regions. In all the SNR levels, CRP model outperforms the others in term of f-score (overall performance). When the SNR level decreased (noise level increased), the overall performance (f-score) of the other software packages decreased more rapidly than CRP, which indicates the CRP model is more robust to noise.

#### Results on Lai's data

**Figure 7:**
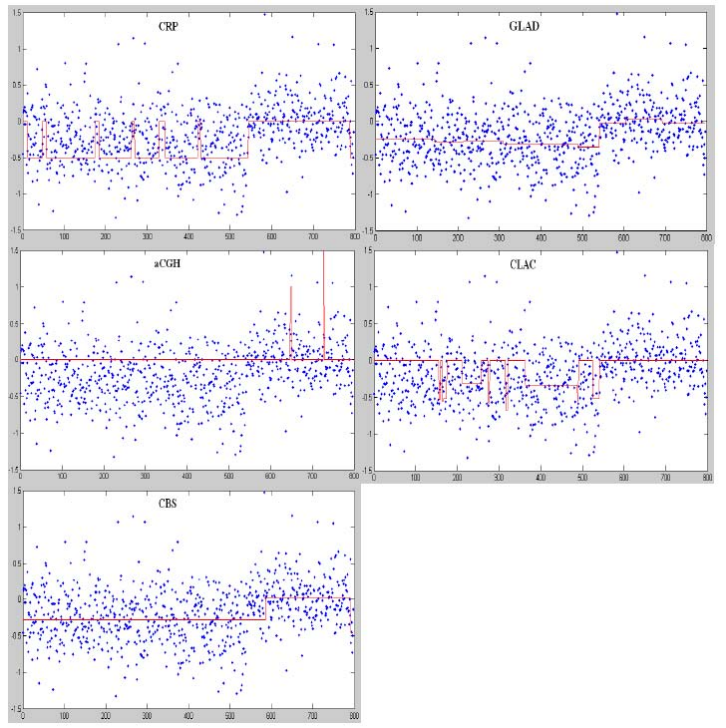
**CNA detection results of five different copy number inferring software packages on Chromosome 13 in a Glioblastoma Multiforme sample (GBM31) used in Lai's 2005 paper**.

The Array-CGH data of chromosome 13 in a Glioblastoma Multiforme sample (GBM31) used in Lai's bioinformatics paper (Lai, et al., 2005) were also tested. Figure 7 provides the CNA region detection results of CRP model and the four widely used software packages. We empirically set the means and standard deviations of CRP as: [-0.27, 0, 0.27] and [0.1, 0.1, 0.1]. As can be seen in Figure 7, all the five methods detected the copy number loss region, whereas the aCGH method wrongly segmented the normal region into the loss region. The CLAC method segmented some loss regions as normal regions. Both CBS and GLAD segmented the whole region into two parts, suggesting that these two methods suppressed noise well and could detect the difference between two chromosomal regions. However, their strong ability to suppress the noise also results in the missing of small CNA regions, as seen in the above results on the simulated data. Additionally, these two software packages only can give the average values of regions rather than the exact the copy number. In contrast, the CRP model generated the exact copy number and detected the major part of the loss region, although a few small loss regions were separated into normal regions.

## Discussion and Conclusions

In this study, we proposed a CRP model for CNA detection, which explores more contextual cues to generate more accurate results than existing methods. The experimental results using both simulated data and real MDS SNP array data show that the CRP model is more robust and reliable compared to a number of widely used CNA detection methods.

For different studies and different noise levels, different number of nodes in the CRP model may be required. More nodes mean more contextual information to be considered, and can suppress higher noise levels, while it is prone to missing of small CNA regions. Fewer nodes will be more sensitive to small CNA regions, and as such be more sensitive to noise. To make the model adjustment convenient, we implemented the algorithm with the number of nodes as a user-input parameter. Thus, it could automatically generate a CRP model with any user-specified number of nodes. The basic idea of generating the CRP model is as follows. First, assume that the shortest CNA region has *m *SNP markers. Then select *m*-1 nearby SNP markers respectively from both the left and right of the current SNP marker. As a result, totally 2*m*-1 (2(*m*-1)+1) SNP markers are selected. In these 2*m*-1SNP markers, there are *m *possible CNA regions with *m *consecutive SNP markers, i.e. drawing *m *consecutive SNP markers out of the total of (2m-1) SNP markers. The rest may be deduced by analogy. There are *m*-1 possible CNA regions with *m*+1 consecutive SNP markers, and to the end, there is only one CNA region with 2*m*-1 SNP markers. Therefore, there are totally  possible CNAs patterns out of these 2*m*-1 SNP markers. In this study, we assume that *m *= 4, then there are 10 possible CNA patterns. In a word, it is convenient for users to test the CRP model with different parameters on their own data.

The parameters in the CRP model were estimated based on some pre-known CNA regions. The users can adjust these parameters according to their specific SNP array data. Also the CRP model gives the probability of each SNP locus staying in each copy number status. If too many CNA regions are detected (may be caused by the bias of the parameters), the users can remove some CNA regions with low probability. Currently, the Haldane's map function is employed as the transition potential in the CRP model. For different diseases and different populations, some prior information of the copy number change can be included into the transition potential. For example, if certain regions of the genome are known to the 'recombination hotspots', then we can set the transition possibility from one copy number to the other higher. Therefore, the future extension of CRP model will consider the SNP-specific copy number aberration rates, and automatically adjust the structure (number of nodes) of the CRP model in running the program.

## Methods

### Log-2-ratio Feature Extraction

**Figure 8:**
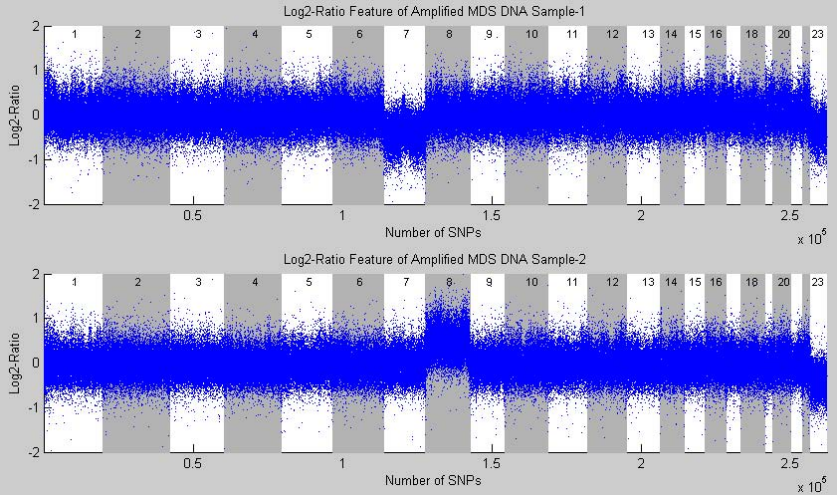
**Log-2-ratio features of two GWA-amplified MDSs DNA samples**.

Due to the high variability of the mean intensities across different SNP arrays, normalization is necessary to make different SNP arrays comparable [19]. In this study, we employed the normalization method of CNAG [17]. CNAG implemented a normalization method to compensate for the different PCR conditions (length and GC content) to reduce the WGA-induced noise [17], which also incorporated the baseline correction procedure by "setting the chromosome numbers in accordance with the ploidy information, e. g. the cytogenetics or FISH [17]." In summary, let  denote the normalized (with the same means of all autosomal SNPs) sum intensity of the i-th SNP locus in the *k-*th array. Then the log-2-ratio features, , are extracted using the 'best-fit' model [17], where denotes the average of m best-fit references for the *i-*th SNP locus; and *m *is the number of selected reference samples. We assume that reference samples mostly have two copies. The reference DNA samples are collected from the non-tumor derived tissues of MDS patients. Figure 8 provides the representative log-2-ratio features of two amplified MDSs DNA samples (using Affymetrix GeneChip Human Mapping 250 K Nsp SNP array). In sample 1, Chromosome-7 has only one copy (monosomy), and Chromosome-8 has three copies (trisomy) in sample 2.

### Copy Number Inference

In the second tier, the statistical model borrows the contextual information to suppress the noise. In this section, we propose a CRP model that extends the use of copy number dependency.

#### Graphical Model of CRP

**Figure 9:**
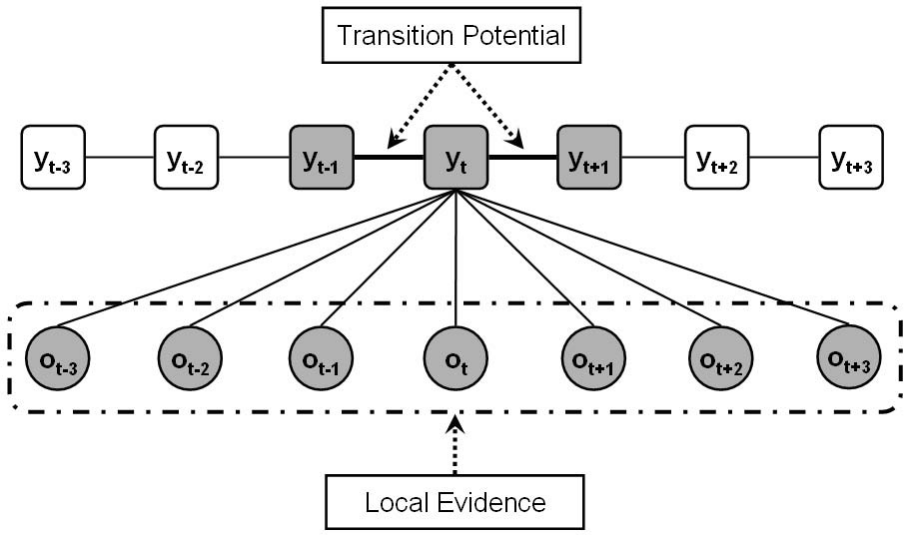
**Partial graphical structure of the CRP model, where *o *denotes the observations and *y *denotes hidden copy number states**.

Figure 9 presents the partial graphical structure of the CRP model. These directly connected hidden states *y*_*t*-1_, *y*_*t*_, *y*_*t*+1 _and observations *x*_*t*-3_, *x*_*t*-2_, *x*_*t*-1_, *x*_*t*_, *x*_*t*+1_, *x*_*t*+2_, *x*_*t*+3 _constitute a maximum clique [44] at instant *t*. To infer the current hidden state, *y*_*t*_, all the vertices in the maximum clique will be used. In other words, the current hidden state is not only determined by its immediate previous and next hidden states, but also several previous and subsequent observations. In the maximum clique, we call the edges between the hidden states as transition potentials, and the edges between hidden states and observations as local evidence. Transition potentials penalize change of hidden states, while local evidence provides the possibility of assigning a copy number to current hidden state.

#### Mathematical Formulation

**Figure 10:**
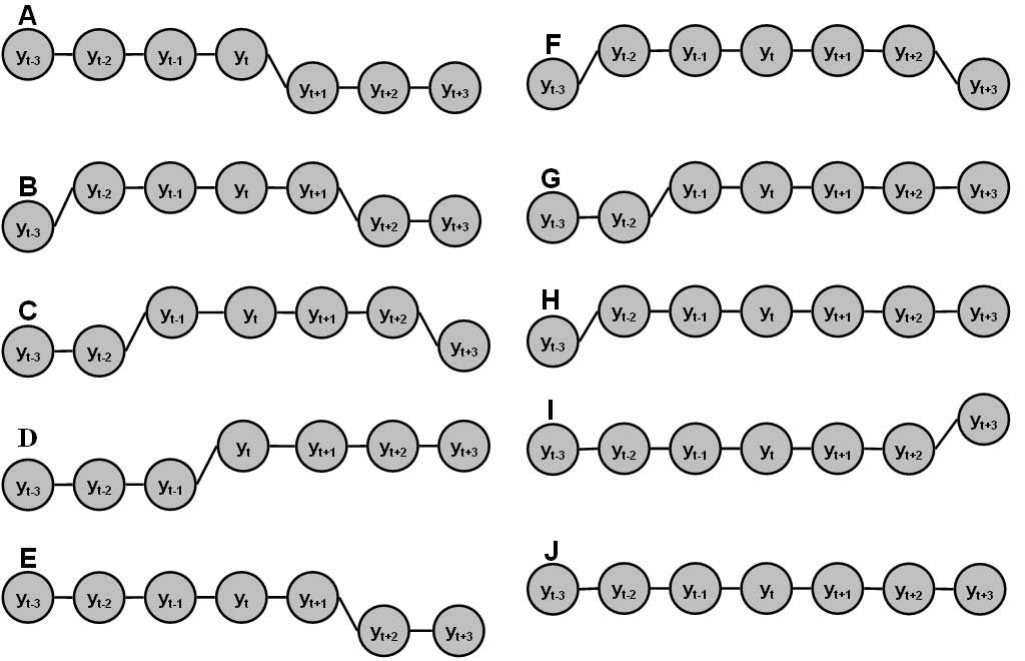
**Ten possible copy number dependency patterns in seven continuous SNP loci under the assumption that at least four continuous SNPs in the CNA regions**. The then possible patterns are as follows. A: *y*_*t*-3 _= *y*_*t*-2 _= *y*_*t*-1 _= *y*_*t*_, *y*_*t*+1 _= *y*_*t*+2 _= *y*_*t*+3_, and *y*_*t *_≠ *y*_*t*+1_; B: *y*_*t*-2 _= *y*_*t*-1 _= *y*_*t *_= *y*_*t*+1_, *y*_*t*-3_, ≠ *y*_*t*-2_, *y*_*t*+1* ≠ *_*y*_*t*+2_, *y*_*t*+2 _= *y*_*t*+3; _C: *y*_*t*-1 _= *y*_*t *_= *y*_*t*+1 _= *y*_*t*+2_, *y*_*t*-3_, = *y*_*t*-2_, *y*_*t*-2 _≠ *y*_*t*-1 _and *y*_*t*+2 _≠ *y*_*t*+3; _D: *y*_*t *_= *y*_*t*-1 _= *y*_*t*+2 _= *y*_*t*+3_, *y*_*t*-3_, = *y*_*t*-2_, *y*_*t*-1_, and *y*_*t*+1 _≠ *y*_*t*; _E:*y*_*t*-3_=*y*_*t*-2_=*y*_*t*-1_=*y*_*t*_=*y*_*t*+1_, *y*_*t*+1 _≠ *y*_*t*+2 _and *y*_*t*+2_=*y*_*t*+3 _; F:*y*_*t*-2_=*y*_*t*-1_=*y*_*t*_=*y*_*t*+1_=*y*_*t*+2_, *y*_*t*-3 _≠ *y*_*t*-2 _and *y*_*t*+2 _≠ *y*_*t*+3 _;G: *y*_*t*-1_=*y*_*t*_=*y*_*t*+1_=*y*_*t*+2_=*y*_*t*+3_, *y*_*t*-3_=*y*_*t*-2 _and *y*_*t*-2 _≠ *y*_*t*-1 _; H: *y*_*t*-2_=*y*_*t*-1_=*y*_*t*_=*y*_*t*+1_=*y*_*t*+2_=*y*_*t*+3_, and *y*_*t*-3 _≠ *y*_*t*-2 _; I: *y*_*t*-3_=*y*_*t*-2_=*y*_*t*-1_=*y*_*t*_=*y*_*t*+1_=*y*_*t*+2_, and *y*_*t*+2 _≠ *y*_*t*+3 _; J: *y*_*t*-3_=*y*_*t*-2_=*y*_*t*-1_=*y*_*t*_=*y*_*t*+1_=*y*_*t*+2_=*y*_*t*+3_.

In CRP model we define the conditional probability, *p*(***y***|***x***), as following:(1)

where, **y **is the inferred copy number sequence; **x **is the observed log-2-ratio feature sequence, and Z(x) = ∑_y _exp(*ψ*(**y'**, **x**)) is a normalization term. The transition potentials and local evidence, as seen in the edges in Figure 10, are integrated in *ψ*(**y**, **x**) as:(2)

where,  is the indicator function, *f*_*TP*_(*y*_*t*-1_, *y*_*t*_) is a transition potential function, and *f*_*LE *_(*x*_*t*-3_, *x*_*t*-2_, *x*_*t*-1_, *x*_*t*_, *x*_*t*+1_, *x*_*t*+3_, *x*_*t*+3_, *y*_*t*) _is the local evidence function; *T *is the number of SNPs (there are 262264 SNP loci in GeneChip Human Mapping 250 K Nsp SNP array), and *K *is the number of considered copy numbers in the model, for example if the considered copy number set is 0, 1, 2, 3, 4}, then *K *= |{0, 1, 2, 3, 4}| = 5.

In the CRP model, we employ Haldane's map function [19,45], which is used to describe genetic distances and recombination fractions, as transition potential function, which can be written as follows:(3)

where , and *d *(unit: 1 Million base-pairs) is the genome distance between two consecutive SNP loci. This function is used to take into account of distances between markers, and indicates that the nearby SNPs are more likely to have the same copy number, whereas the distant SNPs do not [19]. For different diseases, the prior information of the copy number change can be included into the transition potential. The genome distance information can be found at the Affymetrix website http://www.affymetrix.com/index.affx.

#### Local Evidence Function

In the CRP model, we use seven continuous (log-2-ratio) observations to infer copy number of current hidden state, *y*_*t*_, as seen in Figure 9. The reason for considering seven observations is that only the CNA regions that continue *at least *four continuous SNP loci are reliable due to the high noise level. It is straightforward to extend the CRP model to fewer or more SNP loci. Based on above assumption, given seven continuous SNP loci, there are ten possible copy number dependency combinations, as seen in Figure 10. Based on the ten possible dependency combinations, we define the local evidence function as follows.(4)

where , is the probability of copy number *y*_*t *_emitting the observation . The ten terms in the right of Equation (4) are derived from the above ten combinations. In combination A, for example, the observations, *x*_*t*-3_, *x*_*t*-2_, *x*_*t*-1 _and *x*_*t*_, are emitted from the same copy number status, whereas observations, *x*_*t*+1_, *x*_*t*+2 _and *x*_*t*+3_, are generated by another and different copy number status. Therefore we should use *x*_*t*+3_, *x*_*t*+2_, *x*_*t*+1 _and *x*_*t *_to estimate the supporting evidence for *y*_*t *_as *p(x*_*t*-3_, *x*_*t*-2_, *x*_*t*-1_, *x*_*t *_| *y*_*t*_). Under the assumption of independence of observations, we deduce that . The other terms can be explained similarly. Since these ten terms in Equation (4) are not in the same scale, we re-scale them using the indexical transformation. Suppose the real copy number status of seven continuous SNPs is the same as the combination A, then the likelihood functions, *p*(*x*_*t*-3 _| *y*_*t*_), *p*(*x*_*t*-2 _| *y*_*t*_), *p*(*x*_*t*-1 _| *y*_*t*_) and *p*(*x*_*t *_| *y*_*t*_), will be much larger than *p*(*x*_*t*-1 _| *y*_*t*_), *p*(*x*_*t*-2 _| *y*_*t*_) and *p*(*x*_*t*-3 _| *y*_*t*_). In other words, combination A yields higher evidence score than combinations B, C and D. Combination E, F, G, H, I and J consider the case that the CNA regions contain more SNPs. We reason that the re-scaled term that corresponds to the real underlying copy number status yields the maximum support evidence (likelihood) value. This is why we choose the maximum value as the local evidence.

For the conditional probability, , we assume that it follows a Gaussian distribution as:(5)

**Figure 11:**
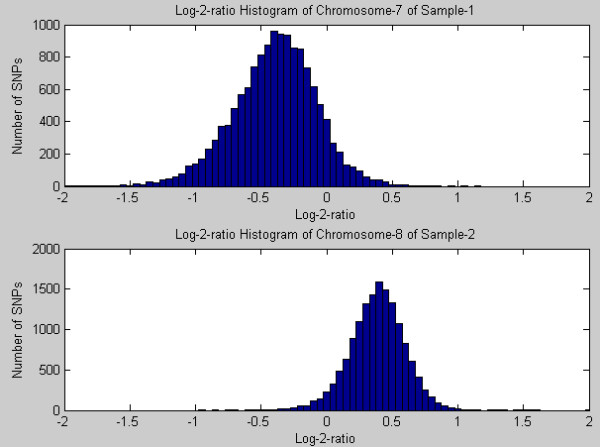
**Histograms of Log-2-ratio features of Chromosome-7 in MDS sample 1, and that of Chromosome-8 in MDS sample 2**.

Where *μ*_*i *_and *σ*_*i *_denote the mean and standard deviation of observed log-2-ratio features emitted by the copy number *i*. We choose the Gaussian distribution according to the investigation of the real data. Figure 11 provides the representative log-2-ratio histograms of known trisomy and monosomy chromosomes of the MDSs data. The means and standard deviations of these Gaussians are estimated using the pre-known CNA regions, e.g. the monosomy and trisomy regions.

#### Model inference

Given a log-2-ratio feature sequence **x**, copy number sequence can be inferred as [41,46]:(6)

The best copy number sequence, , can be obtained using Viterbi algorithm [41,47]. Let *δ*_*t *_(*y*) denote the probability of the best labeling copy number sequence that ends at the *t*-th SNP locus with the label *y*. By induction, we have(7)

The copy number sequence can be obtained by tracing back from *δ*_*T*_(*y*) to *δ*_1_(*y*). The normalized probability of the best labeling  is given by max_*y *_*δ*_*T*_(*y*)/Z(*x*). We implemented the CRP model using Matlab 7.0 based on the conditional random field toolbox, CRFall, written by Kevin Murphy, which can be found at: http://www.cs.ubc.ca/~murphyk/Software/CRF/crf.html. The CRP model has the same asymptotic computational complexity as HMM.

## Authors' contributions

FL participated in the method design, performed the experimental validation and drafted the manuscript. XZ participated in the study design and revised the manuscript. WH acquired the data and performed data analysis. CCC participated in study design and manuscript revision. SW participated in the study design, manuscript revision. All authors read and approved the final manuscript.

## Supplementary Material

Additional file 1 Description of materials, genotyping method and quantitative PCR validation. Click here for file
