# Supplementary material for: Conditional random pattern model for copy number aberration detection
Source: BMC Bioinformatics. 2010 Apr 22;11:200. doi: 10.1186/1471-2105-11-200 (PMC2876128; doi:10.1186/1471-2105-11-200)
Supplement: Additional file 1 — Description of materials, genotyping method and quantitative PCR validation. [file 1471-2105-11-200-S1.DOC]

**Materials**

Twelve cryopreserved bone marrow samples from MDS patients (6 low-grade: refractory anemia (RA) or RA with ringed sideroblasts, 1 intermediate grade: refractory cytopenias and multilineage dysplasia, 4 high-grade: RA with excess of blasts and 1 therapy related-MDS, detailed clinical information in Table 1) were thawed and resuspended into RPMI. The cells were then fractionated into erythroid (CD71 bright and CD34-), immature myeloid (CD10- and high side-scatter), blastic (CD34+) and lymphoid fractions (CD45 bright and low side-scatter) using flow cytometry sorting with FACSAria (Becton Dickinson) by protocol established in our laboratory. Genomic DNA from each fraction was extracted by the Qiagen Allprep RNA/DNA Mini Kit (Qiagen, Valencia, CA) and stored at -80˚C. Constitutional/control DNA consisted of buccal mucosa of 4 patients, the lymphoid fractions of 7 patients and one marrow sample without evidence of MDS sorted into blastic, erythroid and lymphoid fractions. The quality and quantity of genomic DNA was assessed by Nanodrop ND -1000 spectrophotometer (NanoDrop Technologies, Wilmington, DE). Approval of using these samples was obtained from Institutional Review Boards of participating institutions.

**Genotyping method**

Genotyping was performed using 250K *Nsp*I SNP-microarray chips (Affymetrix, Santa Clara, CA) and processed according to the manufacturer’s instruction. The Affymetrix 450 fluidics station and the Affymetrix GCS3000 gene scanner were used to wash, stain and scan the arrays. Signal intensity was analyzed and SNP calls determined using Affymetrix Genotyping Console Version 2.0.

**Quantitative PCR validation**

The copy number of *FOXP2* gene located at chromosome 7q31.1 was further validated by using the real-time quantitative polymerase chain reaction (PCR) assay using the primers for the actin (a housekeeping gene, forward primer 5' TGACAGCAGTCGGTTG 3' and reverse primer 5'GCATTACATAATTTACACGAAAGC 3') and *FOXP2* gene (forward primer 5' CCCTCGGTATGTTAGACAC 3'and reverse primers 5'ACCTCAAACCACGAGT 3 ' sequences) with the LightCycler FastStart DNA Master SYBR Green Quantification Kit (Roche Applied Science, Indianapolis, IN). All reactions were performed in triplicate. The copy numbers of each gene were determined used established standard curves for each gene using method slightly modified from previously described work [1,2]. The ratio of *FOXP2* gene copy number to actin copy gene number in each fraction were then determined. The ratio of each fraction was then normalized to the lymphoid fraction which should have ratio of one to determine the gain or loss of *FOXP2*.

**References**

[1] Chang, C.C., Bredeson, C., Juckett, M., Logan, B. & Keever-Taylor, C.A. (2003) Tumor load in patients with follicular lymphoma post stem cell transplantation may correlate with clinical course. *Bone Marrow Transplant,* **32,** 287-291.

[2] Larionov, A., Krause, A. & Miller, W. (2005) A standard curve based method for relative real time PCR data processing. *BMC Bioinformatics,* **6,** 62.
